# The Kindness Cure

**Published:** 1919-10-18

**Authors:** Ronald Campbell Macfie


### THE KINDNESS CURE.

By RONALD CAMPBELL MACFIE, M.A., M.B., C.M., LL.D.

The great majority of hospital neurasthenics are unhappy, and the unhappiness is frequently due not so much to the neurasthenia itself, as to the dislocations, deprivations, and disabilities the neurasthenia has occasioned in their lives. Headache, weariness, insomnia, lack of power of concentration are afflictions, but men may have all these troubles, and yet remain radically happy. A hospital neurasthenic has all these afflictions of the flesh to bear, and in addition he has to endure banishment from his home, and from his usual advocations and avocations. He has been evicted from his former mental and emotional environment; his life has been dislocated; the limbs of his energies have been put out of joint; and even if he have not painful memories behind and grievous anxieties ahead, he cannot easily adjust himself to a new and cramped physical and psychical milieu. He is like a bishop set by Bolshevists to paint his own palace. He is like a retired business man who cannot make his garden replace his office or literature compensate for his ledger.

Undoubtedly neurasthenia renders it much more difficult for a man to re-orient himself and adapt and adjust himself to unfamiliar conditions, and undoubtedly, too, the neurasthenia aggravates the unhappiness normally consequent on failure in adaptation, but there is a vicious circle, for the unhappiness also aggravates the neurasthenia, and make adaptation doubly difficult. If one, therefore, can make a neurasthenic happier in his outlook on life one can certainly facilitate and hasten his recovery from his neurasthenia.

To a certain extent this is recognised at all nerve hospitals; and the nerve patient is provided with entertainments and occupations—books, music, lectures, excursions, theatres. With all these he is provided, and yet he is made little happier by them, for these really help him very little to adapt himself to the realities of his present and his future. It is not enough to offer a neurasthenic patient an hour's distraction; *that* has its uses; but it is not distraction he requires so much as integration and reconstruction. His whole daily life has to be reconstructed and reinspired, till, in spite of its handicaps and limitations, it seems worth living; his whole attitude towards life has to be made patient, and tolerant, and cheery; and this can be effected only by personal influence, and understanding sympathy. A patient must be helped to make the most and the best of existence; he must be helped to adapt himself to new and difficult conditions; and nothing will heal his soul and promote his happiness more than real kindness, and friendship, and sympathy from those around him.

If a patient has an environment of friendship, and if those around him take an interest in him not only as a neurasthenic case but also as a human being—if they take a friendly interest in his past, and present, and future, in his family, in his dreamings and doings, then gradually much of his unhappiness will go, for he will feel that the world has got kindness in it, and that even in spite of headaches and insomnia, it is not altogether a bad place to live in.

It is not enough to set a man to make boots; we must take an interest in the boots he makes, and suggest that they are worth making, otherwise the neurasthenic's own interest will quickly flag. It is not enough to advise a patient to garden; we must see to it that he takes an interest in his gardening, and we can best quicken his interest by sharing it.

In hospitals, nurses and doctors are apt to forget that neurasthenic patients require to be encouraged to face life, as well as encouraged to do work or to eat their dinner. A patient who leaves hospital sleeping better, eating better, altogether better than on admission, but nevertheless discontented, unhappy, and misanthropic, is much more likely to relapse under the strain of life than a patient discharged in poorer health who has learned to take a happier view of existence.

Pity and coddling are to be abjured; they will only breed self-pity and futility; but genuine personal interest and friendliness are of the greatest value in the treatment of neurasthenia.

Lately we heard a sister in a nerve hospital boasting herself to be a "splendid disciplinarian." Discipline is a good thing; discipline is a necessary thing; but a sister who carries on her work in the spirit of a splendid disciplinarian, and who boasts of it, is not the right sister to deal with neurasthenic patients.

